# Late Oral Complications in Childhood Cancer Survivors: Implications for Pediatric Dentistry and Survivorship Care

**DOI:** 10.3390/children13010114

**Published:** 2026-01-13

**Authors:** Lucija Ruzman, Ana Zulijani, Tomislav Skrinjaric, Domagoj Buljan, Jasminka Stepan Giljevic, Iva Bilic Cace, Ana Milardovic

**Affiliations:** 1 Department of Pediatrics, School of Medicine, University of Rijeka, 51000 Rijeka, Croatiaana.milardovic@medri.uniri.hr (A.M.); 2 Department of Dental Medicine, Faculty of Dental Medicine and Health, Josip Juraj Strossmayer University of Osijek, 31000 Osijek, Croatia; azulijani@fdmz.hr; 3 Department of Oral Surgery, Clinical Hospital Centre Rijeka, 51000 Rijeka, Croatia; 4 Department of Pediatric and Preventive Dentistry, School of Dental Medicine, University of Zagreb, 10000 Zagreb, Croatia; 5 Clinical Department of Pediatric and Preventive Dentistry, Dental Clinic, University Hospital Center Zagreb, 10000 Zagreb, Croatia; 6 Department of Oncology and Hematology, Children’s Hospital Zagreb, 10000 Zagreb, Croatia; domagoj.buljan@kdb.hr (D.B.);; 7 School of Medicine, University of Zagreb, 10000 Zagreb, Croatia

**Keywords:** cancer survivors, child, long-term adverse effects, long-term care, oral health

## Abstract

**Highlights:**

**What are the main findings?**

- Childhood cancer survivors frequently experience late oral complications, which remain underrecognized and insufficiently monitored.
- Children treated at an early age, those receiving head and neck radiotherapy and those exposed to intensive chemotherapy are at the highest risk, as these interventions can permanently impair dentofacial development.

**What are the implications of the main findings?**

- Standardized, evidence-based guidelines for long-term follow-up and management of oral complications need to be developed to ensure timely diagnosis and intervention.
- In survivorship care, dentists should be integrated as equal members of the multidisciplinary team to ensure optimal long-term follow-up.

**Abstract:**

Survival rates for children treated for malignant diseases continue to improve, yet many survivors face persistent late oral complications that affect function, aesthetics, and quality of life. Oncological therapy, especially at a young age and following head and neck radiotherapy or intensive chemotherapy, can disrupt dental and craniofacial development, resulting in dental developmental disorders, enamel defects, salivary gland dysfunction, caries susceptibility, periodontal problems, trismus, and osteoradionecrosis of the jaw. Although these effects are partially known, they are frequently underrecognized in routine practice, and many children do not receive adequate long-term dental follow-up. A key challenge highlighted in the recent literature is the absence of structured, evidence-based guidelines for monitoring and managing late oral effects. The article emphasizes the need for clearer recommendations, better communication of oncological treatment histories, and stronger integration of dental professionals within survivorship care. Developing standardized follow-up protocols will be essential to ensure timely detection, consistent management, and improved oral health outcomes for childhood cancer survivors. This article is intended as a narrative review, synthesizing available evidence from key publications to highlight clinically relevant late oral complications and gaps in current survivorship care.

## 1. Background

Malignant diseases in children and adolescents are rare, accounting for less than 1% of the total cancer patient population. In Europe, the average age-standardized incidence rate of malignant diseases in children aged 0–14 years is between 135 and 150 cases per million people per year, while rates of 170–200 cases per million are recorded in adolescents aged 15–19 years [1]. Pediatric malignancies differ substantially from adult cancers in tumor type, biology, etiology, and therapeutic strategies. Pediatric malignancies are most frequently of hematological origin and often result from genetic alterations that may occur prenatally, whereas tumors in adults are more commonly associated with accumulated environmental and lifestyle exposures. The most prevalent childhood malignancies are leukemias, with acute lymphoblastic leukemia (ALL) being the most common, central nervous system tumors, lymphomas, neuroblastomas, and bone and soft tissue sarcomas. Less common tumors affect the liver, kidney, retina, and germ cells. Although children typically exhibit a more favorable response to aggressive treatments such as chemotherapy and radiotherapy and achieve higher five-year survival rates, the acute and long-term side effects of therapy are more pronounced due to ongoing growth and developmental processes [2]. With cure rates of 80–90% in developed countries [1], the care of pediatric cancer patients is increasingly focused on monitoring late treatment effects. This excellent success of oncological and supportive treatment of children and adolescents with malignant diseases has resulted in an increasing population of survivors, collectively referred to as Childhood Cancer Survivors (CCS). It is estimated that in Europe, 300,000 to 500,000 people (1:1000 of the general population) were treated for malignant disease in childhood or adolescence [3]. Approximately two-thirds of CCS experience at least one late complication [4], which may affect various organ systems, including the oral cavity. These late complications are often underrecognized in clinical practice and are frequently inadequately addressed in routine survivorship care. Timely identification of such complications can influence treatment strategies, reduce late morbidity, and ultimately improve quality of life. Therefore, systematic, long-term follow-up (LTFU) care for this population is needed [5], adapted to each individual’s age and needs. Although awareness of late oral complications resulting from oncological treatment is increasing, clinical guidelines for their prevention, monitoring, and treatment remain lacking.

## 2. Review Methodology

Literature relevant to the topic was identified through electronic database searches of PubMed, Scopus, Google Scholar, and Web of Science, with the search limited to studies published within the past ten years (2015–2025). Searches were conducted using the following keywords/Medical Subject Headings (MeSH) terms and their combinations: “Child” [MeSH], “Adolescent” [MeSH], “Pediatrics” [MeSH], “Cancer Survivors” [MeSH], “Neoplasms” [MeSH], “Drug Therapy” [MeSH], “chemotherapy”, “Radiotherapy” [MeSH], “head and neck irradiation”, “Stem Cell Transplantation” [MeSH], “dental developmental disorders”, “Tooth Abnormalities” [MeSH], Developmental Defects of Enamel [MeSH], “Xerostomia” [MeSH], “hyposalivation”, “Dental Caries” [MeSH], “Periodontal Diseases” [MeSH], “Trismus” [MeSH], “Osteoradionecrosis” [MeSH], “Graft vs. Host Disease” [MeSH], “subsequent primary malignancies”, “late oral complications”, “Oral Health” [MeSH], “survivorship care”, “Long-Term Care” [MeSH], and “Long-Term Adverse Effects” [MeSH]. Boolean operators (AND, OR) were used to optimize the search, and MeSH terms were applied where appropriate to enhance precision. The search was restricted to English-language articles, and additional articles were identified by manually screening the reference lists of selected studies. Conference abstracts, unpublished studies, and articles not directly related to primary research objectives were excluded from the review. A total of 96 articles were included based on their relevance, scientific rigor, and contribution to understanding the interplay between childhood cancer treatment, late oral complications, and long-term survivorship care. Of these, 58 original research articles and nine review articles directly addressed late oral complications and survivorship care in CCS, whereas the remaining studies provided supportive clinical, biological, or methodological context. Formal inclusion/exclusion criteria and a structured quality assessment were not applied; however, all included studies were critically appraised for methodological quality, population size, diagnostic criteria, and potential confounding factors. Given the heterogeneity of study designs and outcomes, a narrative synthesis approach was considered the most appropriate.

In contrast to previous reviews that have primarily addressed dental developmental disorders (DDD) or the prevalence of specific oral sequelae, this narrative review offers a comprehensive, clinically focused synthesis of late oral complications in CCS. It integrates dental, periodontal, salivary, functional, and survivorship-related aspects, emphasizing interdisciplinary care and the importance of LFTU.

## 3. Pathophysiology of Late Oral Complications

Odontogenesis and amelogenesis are intricate processes involved in tooth and enamel development that require precise interaction between the epithelium and mesenchyme. Disruptions in these processes, whether due to genetic, nutritional, hormonal factors, or therapeutic interventions such as chemotherapy or radiation, may result in permanent DDD [6]. Because ameloblasts and odontoblasts are highly sensitive cells, any impairment of their function can lead to hypoplasia, hypocalcification, or other dental abnormalities.

Chemotherapy targets tumor cells but also affects other rapidly dividing cells. Unlike radiation, which is confined to the treatment area, chemotherapy produces systemic effects. As a result, odontogenic cells located far from the tumor site may also be damaged. Because chemotherapeutic agents have short half-lives, dental defects typically arise from temporary disruption of odontoblast function rather than cell death, resulting in primarily localized changes [7,8].

An extensive epidemiological study conducted in 2009 demonstrated that radiation to the dentition and jaw region, particularly at doses exceeding 20 Gy, and high-dose alkylating chemotherapy administered at an early age independently and significantly increase the risk of late oral complications in CCS [8]. Chemotherapeutic agents, including vincristine, vinblastine, and cyclophosphamide, may induce hypomineralized enamel defects by disrupting the calcium transport mechanism in ameloblast microtubules [7,9]. Specific chemotherapeutic agents, such as vinblastine and vincristine, may damage mature odontoblasts and ameloblasts. These agents disrupt microtubule function in odontoblasts, thereby impairing collagen fiber formation and dentin matrix formation. This disruption leads to shortened, thin, and narrowed tooth roots [7]. Intensive and repetitive chemotherapy administered during the initial phase of hard-tissue formation may result in tooth agenesis [10]. A study by Kang CG et al. [11] showed that children who received cisplatin- or carboplatin-based chemotherapy had a high risk of severe dental anomalies, and this risk was particularly pronounced when they received ≥4 classes of chemotherapeutic agents.

Radiation impairs tooth development by directly inhibiting odontoblast division and, indirectly, by stimulating osteodentin formation, which replaces normal dentin. Because osteodentin contains fewer phosphorylated phosphoproteins involved in enamel crystal nucleation, this results in reduced mineralization and an irregular enamel structure [12]. Radiation therapy administered to the head or neck typically involves doses ranging from 27 to 70 Gy, which can significantly disrupt normal craniofacial growth [13]. Furthermore, radiation exposure in this area is the primary predictor of dental damage [14], with late oral complications reported in approximately 60% of patients receiving radiotherapy [15]. The risk increases with the use of concomitant chemotherapy and is further elevated by higher radiation doses and younger age at exposure [14]. Inclusion of the jaw within the radiation field may delay or alter maxillary and mandibular development, leading to facial asymmetry and functional impairments. Additionally, radiotherapy, especially when both parotid glands are affected, can cause permanent damage to acinar cells and impair salivary gland function by reducing saliva volume, increasing viscosity, lowering pH, and diminishing remineralization capacity [16,17,18]. It may also induce trismus through fibrosis of the masticatory muscles and temporomandibular joint, and increase the risk of osteoradionecrosis of the jaw (ORNJ) [14,19].

The timing of oncological treatment, especially when administered before the age of five, a period characterized by rapid odontogenesis and amelogenesis, is critical for the development of oral complications [14,15,20,21,22,23]. The impact of oncological therapy on late oral complications is even significantly greater when treatment was administered before age three [11]. Such dental disturbances may result in functional, aesthetic, and psychosocial challenges, frequently necessitating prolonged and complex dental management. Additionally, treatment during this vulnerable developmental stage can substantially impair salivary gland function, craniofacial growth, and temporomandibular joint development, thereby further compromising oral health outcomes in very young individuals [19].

While the dental consequences of chemotherapy in the CCS population are well documented, distinguishing between effects attributable solely to chemotherapy and those resulting from combined chemoradiotherapy remains challenging [15]. The specific cumulative doses of individual cytostatic agents responsible for distinct levels of dental damage remain undetermined, and several risk factors have yet to be identified. Furthermore, the chemotherapeutic agents that most significantly contribute to the development of these damages have not been clearly established. Moreover, the radiation doses that cause damage to the salivary glands or masticatory muscles, or induce osteonecrosis of the jaw, have not yet been clearly defined [17].

## 4. Late Oral Complications of Childhood Cancer Treatment

Cancer treatments such as chemotherapy, radiotherapy, immunotherapy, and hematopoietic stem cell transplantation (HSCT) can have a significant long-term impact on oral health. The most prevalent complications include dental caries and DDD, such as enamel hypoplasia, root shortening, and dental agenesis. Furthermore, CCS exhibit a higher incidence of hyposalivation and increased colonization by cariogenic microorganisms, thereby elevating the risk of periodontal disease and caries. Subsequent primary malignancies (SPM) within irradiated fields may significantly compromise oral health and overall well-being in pediatric patients. Additional late effects include chronic graft-versus-host disease (cGVHD) of the oral cavity, trismus, and ORNJ. Dentists and pediatric oncologists should recognize that early-age treatment may delay the manifestation of dental abnormalities until years post-therapy, despite effective disease control. Research based on self-reported dental difficulties, derived from a single study, demonstrates that nearly half of CCS patients experience at least one late oral complication [24], a finding corroborated by objective assessments. Dental examinations confirm that late oral complications are present in approximately half of CCS [15,22]. Nevertheless, late oral complications remain underrecognized in clinical practice and frequently go untreated, despite their substantial impact on long-term health and quality of life.

### 4.1. Dental Developmental Disorders

Chemotherapy and radiotherapy can disrupt normal root development, leading to shortening, known as “root stunting”. Other DDD, such as microdontia (reduced tooth size), hypodontia (lack of permanent teeth), and prolonged retention of primary teeth, are also common [8,25]. Studies have demonstrated a higher prevalence of hypodontia, microdontia, and shortened dental roots in children and adolescents receiving chemotherapy for ALL compared to healthy controls [20,26,27,28,29,30]. Similar DDD has been observed in pediatric patients with neuroblastoma [20,31,32], rhabdomyosarcoma [33,34,35], nephroblastoma [30,36], brain tumors [27,37,38], as well as lymphoma and other solid tumors [22,39].

A meta-analysis showed that children receiving oncology therapy had twice the risk of at least one DDD (relative risk, RR = 2.00) and almost nine times the odds compared with healthy controls (odds risk, OR = 9.04) [21]. Recent studies showed that 6.1% of CCS reported at least one DDD [40]. DDD occurs in 70–100% of children receiving head and neck radiotherapy, especially those younger than 4 years, and the most severe changes occur when developing teeth receive doses of 20–30 Gy or more [17]. Multiple studies have confirmed the importance of age, consistently showing a higher incidence of DDD among children who receive treatment at younger ages [14,15,21,25].

In addition to aesthetic consequences, these changes can cause functional difficulties, including malocclusion, reduced chewing ability, temporomandibular joint problems, and slurred speech [41]. Consequently, many CCS with DDD not only require comprehensive orthodontic therapy in adolescence [42] but also additional procedures, such as endodontic treatment, restorative procedures, and extended functional rehabilitation.

### 4.2. Enamel Abnormalities

Oncological therapy may disrupt ameloblast function during enamel formation, leading to enamel hypoplasia (pits, grooves, and enamel defects), opacities (white, yellow, or brown spots of varying sizes and intensities), and discoloration [43]. The prevalence of enamel hypoplasia among CCS has been reported to range from 3% to 58% [14,15,17,23,26,29,39,44], while opacities and discolorations have been documented in 12% to 68% of survivors [23,39,44,45]. The risk is higher with combined treatment protocols. These results suggest that permanent enamel defects are associated with tooth sensitivity, an elevated risk of caries, and aesthetic concerns, underscoring the need for dental LTFU [45,46].

### 4.3. Hyposalivation and Cariogenic Microbial Profile

Hyposalivation is associated with both radiotherapy and chemotherapy. Stolze J et al. [47] found that nearly a third of participants had reduced saliva production, but only 10% reported xerostomia. Reduced salivary flow alters the spectrum of bacteria colonizing the oral cavity, thereby promoting the growth of caries-associated microflora. Avşar A et al. [44] found that children treated with chemotherapy had reduced salivary flow and a higher prevalence of cariogenic bacteria (*Streptococcus mutans* and *Lactobacillus*) compared to healthy peers. Changes in salivary volume and quality can lead to mucosal sensitivity, increased risk of caries and infections, altered taste, and difficulties with eating, swallowing, speaking, and even sleeping [48]. The function of the salivary glands is particularly compromised by radiation therapy, which reduces salivation and lowers pH, thereby favoring the growth of cariogenic bacteria and increasing the risk of caries and periodontal disease [49,50]. Among pediatric patients who received radiotherapy for nasopharyngeal carcinoma, approximately 66.7% experienced xerostomia as a late complication [51]. A recent meta-analysis reported that radiation doses of 35–40 Gy to the parotid glands are associated with xerostomia in up to 32% of CCS, with risk increasing in a dose-dependent manner. Due to insufficient pediatric-specific data, the authors recommend applying adult guidelines and limiting the mean parotid dose to <26 Gy [17].

### 4.4. Periodontal Complications

Periodontal diseases result from the accumulation of pathogenic microbial biofilm (plaque) on and below the gingival margin. This accumulation initiates an immunoinflammatory response in the host, ultimately leading to the destruction of supporting tooth structures, including the gingiva, periodontal ligament, and alveolar bone [52]. The acute sensitivity of periodontal tissues to oncological treatment is well established. However, the long-term periodontal consequences of such therapies have not been sufficiently investigated. Studies on self-reported oral problems indicate that 10% to slightly more than 30% of CCS experience severe gingivitis and/or periodontitis [53,54]. Clinical investigations have confirmed these findings, reporting significantly higher plaque and gingival indices in CCS compared to healthy controls [39,55,56]. Additionally, Longo BC et al. [55] identified significantly greater probing depth (PD) and clinical attachment level (CAL) values, as well as a shift toward periodontopathogenic species, including *Fusobacterium nucleatum*. Timely prevention and treatment of initial periodontal disorders is essential to prevent further loss of connective tissue and alveolar bone, thereby reducing the risk of tooth loss during adolescence and adulthood.

### 4.5. Caries

The most frequently reported late oral effect of oncological treatment is an increased prevalence of dental caries, reflected in higher DMFT/dmft scores (Decayed–Missing–Filled Teeth) [15,30,38,44,45,57,58,59,60,61]. Dental caries is a chronic disease caused by bacteria in the oral cavity that produce acid as a byproduct of metabolizing fermentable sugars in food. Changes in salivary volume and composition, enamel defects, and shifts in the oral microbiome toward more cariogenic microflora are considered significant factors in the increased risk of caries in CCS [62]. Additional risk factors include poor oral hygiene, intake of sugar-based oral solutions, dietary habits, and socioeconomic and demographic circumstances. It is estimated that 20.4% of CCS have an increased susceptibility to caries [47]. Nasim vs. et al. [63] reported that DMFT scores were significantly higher in children who received combined chemotherapy and radiotherapy than in those treated with chemotherapy alone. Dental caries is a preventable dental complication among CCS. However, without prompt intervention, it can result in the loss of enamel and dentin and may advance to involve the pulp and adjacent bone, potentially causing severe complications.

### 4.6. Trismus

Trismus refers to a diminished capacity to open the mouth, commonly identified by a maximum interincisal distance (MID) ≤ 35 mm [64]. In CCS, trismus following chemotherapy and radiotherapy most often results from damage to the masticatory muscles (masseter and pterygoid) and the temporomandibular joint. Radiotherapy represents the predominant etiological factor, as it often leads to fibrosis and muscle contractures. Symptoms typically develop gradually, beginning several weeks to months following the completion of radiotherapy, and are characterized by a progressive reduction in mouth opening over subsequent years [65]. Trismus substantially impairs essential daily functions, including speech, eating, and oral hygiene practices. The prevalence of trismus among patients receiving radiotherapy for head and neck tumors ranges from 5% to 38% in adults [66], with higher rates observed in those exposed to increased radiation doses and involvement of the masticatory muscles. Epidemiological data on trismus in the pediatric population are largely unavailable, except for one article that reports a 7.1% prevalence of trismus as a late complication in children treated for nasopharyngeal carcinoma with combined chemoradiotherapy [67].

### 4.7. Osteoradionecrosis of the Jaw

Osteonecrosis of the jaw (ONJ) is clinically characterized by exposed necrotic bone, or bone that can be probed through a periodontal pocket or an intraoral or extraoral fistula, and persists without healing. ONJ may result from radiation therapy to the head and neck, referred to as osteoradionecrosis (ORNJ) or from the use of antiresorptive or antiangiogenic medications, known as medication-related osteonecrosis of the jaw (MRONJ). Both conditions are rare in pediatric patients. The reported prevalence of ORNJ in the adult population ranges from 5% to 15%, depending on factors such as patient population, radiation dose, dental care, and length of follow-up [68]. Although ORNJ is a well-recognized complication in adults, its incidence and associated risk factors in pediatric patients remain poorly defined. In a single-institutional cohort of 117 pediatric patients treated with proton therapy for head and neck malignancies, ORNJ was uncommon, occurring in only 1.7% of patients, with high radiation doses and prior dental procedures identified as contributing factors [69]. Likewise, only a limited number of reports have investigated MRONJ in the pediatric population. A recent systematic analysis by Rosales HD et al. [70] reported that MRONJ occurs extremely rarely (0.16–1.1%) in pediatric patients treated with antiresorptive or antiangiogenic drugs, even after prolonged therapy and frequent dental procedures. However, this review excluded children who had received oncological treatment involving radiotherapy or chemotherapy. To our knowledge, no published articles or case reports have documented ONJ in CCS.

### 4.8. Chronic Graft-Versus-Host Disease of the Oral Cavity

Chronic graft-versus-host disease (cGVHD) represents a significant late complication of allogeneic HSCT, a procedure commonly employed in the management of high-risk and relapsed hematological malignancies. cGVHD typically develops more than 100 days after transplantation and may persist for years, often affecting multiple organ systems. Oral manifestations are observed in approximately 28–80% of patients with cGVHD [71,72]. Diagnosis relies on clinical examination based on the 2014 National Institutes of Health (NIH) consensus criteria [73], which identify three primary pathological processes in the oral cavity: oral mucosal disease, salivary gland dysfunction, and sclerotic changes. The most frequent clinical features include erythema, lichenoid changes, ulcerations, pseudomembranes, edema, mucosal pain and burning, xerostomia, and restricted mouth opening due to progressive fibrotic alterations [74]. A study by Tanem KE et al. [72] examining oral cGVHD in a younger cohort of allo-HSCT survivors found that xerostomia was present in 26.5% of patients and dysgeusia in 22%. Notably, nearly half of patients exhibited no subjective symptoms despite evident clinical changes [72], indicating that oral cGVHD is frequently unrecognized and often undertreated.

### 4.9. Subsequent Primary Malignancies

CCS, particularly those who have received head and neck radiotherapy, face a significantly elevated lifelong risk of developing SPM in the oral cavity [75,76], including the salivary glands, tongue, pharynx, and other oral structures. This elevated risk is determined by factors such as cumulative radiation dose, size and location of the radiation field, age at exposure, and underlying genetic susceptibility [77]. A significantly elevated risk of salivary gland tumors has been observed, with individuals who received radiotherapy exhibiting a 33-fold higher risk, with the highest incidence occurring among CCS of Hodgkin lymphoma and leukemia [76]. Additionally, individuals with oral involvement of cGVHD are at greater risk for developing oral squamous cell carcinoma [78] (Table 1).

## 5. Gaps and Challenges in Providing Oral Health Care for CCS

A dental consultation for children with newly diagnosed malignancy should be conducted immediately to ensure sufficient time for necessary care prior to the initiation of cancer therapy [79]. An extensive study conducted in the United Kingdom found that only 36% of children with newly diagnosed malignancies received a dental examination prior to treatment [80].

Standardized oral care protocols in pediatric oncology demonstrate high efficacy in preventing oral mucositis during chemotherapy [81]. Although recommendations address the management of mucositis, caries, and xerostomia during treatment, most of them rely on limited evidence. Structured, daily basic oral care remains the only consistently supported intervention [82]. The recommended frequency of tooth brushing varies; however, it should be performed at least twice daily using fluoride toothpaste [80]. Individuals who brush their teeth less than twice daily have a 40–50% increased risk of developing new caries lesions, with this effect being especially significant in primary dentition [83]. The data indicate concern, as only 70% of patients’ parents adhere to clear oral hygiene recommendations, while 30% of children undergoing chemotherapy regularly consume snacks and sweetened beverages; and an additional problem is that the oral solutions that children need to take frequently and for a long time during treatment are sugar-based [84]. Chlorhexidine mouth rinse, in addition to reducing colonization by pathogenic bacteria and fungi [85], is the most frequently used prophylactic therapy for reducing dental plaque and gingivitis, which are crucial for preventing late periodontal consequences. However, the long-term use of these agents is constrained by adverse effects, including tooth staining and alterations in taste [86].

A recently published protocol for oral care in hematological pediatric oncology patients has been proposed for implementation before, during, and after oncology therapy. This approach emphasizes early prevention, ongoing hygiene maintenance, and LTFU to address late oral complications [87]. However, clinical guidelines for the LTFU of late oral complications remain absent.

A significant challenge in the current literature is distinguishing late oral effects that can be attributed exclusively to chemotherapy, radiotherapy, or their combination. This challenge arises because many studies utilize multimodal treatment protocols and overlapping therapeutic exposures. Enhanced understanding of therapy-specific effects would facilitate the development of more targeted prevention and follow-up strategies.

CCS patients are advised to attend dental examinations at least every 6 months and every 3 to 6 months for high-risk CCS [62,87]. Reported dental visit frequencies among the CCS population vary substantially, with around 60% of survivors attending annual dental appointments [88,89], and up to 92% in more recent studies [54]. Socioeconomic status is a significant determinant of oral health. Lower levels of education, reduced income, and limited access to dental care are associated with poorer oral health outcomes. Studies consistently demonstrate that socioeconomic and structural barriers substantially increase the risk of oral disease, particularly among vulnerable populations such as CCS [53,89,90]. Disparities in dental care are typically more pronounced in low- and middle-income countries, where access is often limited, preventive services remain underdeveloped, and curative care is constrained or delayed. Nevertheless, the majority of existing research is derived from highly developed countries, while data from middle- and low-income regions remain limited. A lack of a structured approach is demonstrated by data indicating that 91% of CCS are referred to their primary dentist for LTFU [80], but do not receive specific recommendations for ongoing care.

Studies found that dentists identify only about half of patients’ subjective complaints. For example, xerostomia was noted by 4% of dentists, compared with 9.4% of patient respondents [40]. This discrepancy indicates that dental professionals may often overlook the subjective disturbances experienced by CCS. Among CCS, a lower proportion of filled teeth (F component of the DMFT index) was observed compared with a control group, indicating insufficient access to or implementation of dental care during the recovery phase, as well as potential missed opportunities for caries management and oral health preservation [46]. It is also worth noting that frequent dental specialist examinations and orthodontic procedures impose additional emotional, temporal, and financial burdens on the entire family [91,92], who have already undergone long-term and demanding oncological treatment.

Education of CCS and their caregivers on proper oral hygiene, appropriate nutrition, and the need for regular dental checkups is crucial for preventing and mitigating oral complications during and after treatment. Furthermore, it is crucial to enhance dentists’ awareness of potential late oral complications resulting from oncological treatment and to promote effective preventive strategies.

## 6. Future Directions

Pediatric hemato-oncologists are increasingly focused not only on improving survival rates but also on mitigating long-term treatment toxicity, reducing late complications, and enhancing patients’ quality of life. Raising awareness among other healthcare professionals is crucial for improving interdisciplinary collaboration and outcomes of CCS. Continuous monitoring and management of late complications in CCS is essential.

Patient care should be multidisciplinary, coordinated, accessible, age-appropriate, and holistic. To standardize CCS care, clinical guidelines for late complications of cancer therapy (e.g., cardiotoxicity, infertility, endocrine dysfunction, etc.) have been developed in Europe to promote best practices. PanCare provides expert recommendations [93], while the Children’s Oncology Group (COG) [94] and the International Guideline Harmonization Group (IGHG) [95] offer additional guidance. These guidelines are based on systematic reviews of current scientific evidence. However, despite growing recognition of late oral complications, there remains a lack of scientifically based clinical guidelines for monitoring and managing oral and dental consequences of oncological treatment.

Dental professionals should be informed of each patient’s oncological treatment history, which is a process that will be substantially facilitated once every CCS possesses a personal survivorship passport. The PanCare Survivorship Passport (SurPass), a digital tool implemented in Europe for CCS, provides a comprehensive treatment summary and individualized recommendations for LTFU of late effects [96], with the potential to facilitate more structured and informed dental care.

Taken together, these developments point to several priorities for future directions:

- Development of practical, consensus-based guidelines for monitoring and managing late oral complications in CCS;
- Enhanced integration of dental care within multidisciplinary LTFU programs;
- Assessment of digital survivorship tools in routine dental clinical practice; and
- Improved education for healthcare professionals, patients, and caregivers to support the prevention and early detection of late oral complications.

## 7. Conclusions

The severity of late oral complications is influenced by tumor-related factors, the intensity and modality of anticancer therapy, patient age, pre-existing oral health, and access to appropriate dental care. Referral to a dentist before the initiation of oncology therapy, along with ongoing oral health monitoring throughout treatment and during LTFU, is essential to prevent complications and improve oral and general health outcomes. Effective survivorship care depends on coordinated, interdisciplinary collaboration among dental professionals, pediatric oncology teams, and other healthcare providers. While late oral complications are not always preventable, timely and structured dental care can reduce their impact and support improved long-term health and quality of life.

The findings of this narrative review are limited by its non-systematic methodology, potential selection bias, and heterogeneity among the included studies, driven by variations in study design, patient populations, treatment modalities, and outcome measures.

## Figures and Tables

**Table 1:** Overview of risk factors, late oral complications, and recommendations in childhood cancer survivors.

**Risk factors**	
**Treatment-related factors**	
Chemotherapy (intensive/multimodal)	
Hematopoietic stem cell transplantation	
Head and neck radiotherapy (dose-dependent)	
**Patient-related factors**	
Young age at treatment (<5 years)	
Poor oral hygiene	
Limited access to dental care	
**Major late oral complications**	
Dental development disorders (DDD)	Microdontia [8,10,11,15,17,19,20,21,22,24,25,26,27,28,31,32,34,35,36,37,38,39,40,42,50,53]
	Agenesis [8,10,11,15,17,19,20,21,22,25,26,27,28,31,32,34,35,36,38,39,40,41,42,50,53]
	Enamel defects (hypoplasia/discoloration) [7,8,9,14,15,17,19,20,24,26,29,31,32,35,36,38,39,40,42,43,44,45,46,50,53]
	Root defects (shortened/narrowed/agenesis) [7,8,9,11,14,15,17,19,20,21,22,25,26,27,28,31,32,33,34,35,36,37,39,40,42,44,50,53]
	Delayed eruption/impacted teeth [8,17,19,20,25,28,39]
Dental caries and periodontal diseases	Increased caries risk (higher DMFT) [9,15,17,19,23,24,26,29,30,32,38,44,45,46,49,50,54,58,59,60,61,62,63,67]
	Gingivitis [8,9,19,24,39,46,49,50,53,54,55,56,59,60,61,63]
	Periodontitis [19,24,39,46,54,55,56]
Salivary gland dysfunction	Hyposalivation [14,16,18,19,30,47,48]
	Xerostomia [8,16,17,18,19,35,42,47,48,51,53]
Skeletal and bone complications	Impaired craniofacial growth [19,35,42,50]
	Osteoradionecrosis [19,69,70]
Trismus [19,35,38,67]	Functional limitations (speech/eating/oral hygiene)
Graft-versus-host disease [19,72,74]	Sclerosis
	Lichenoid and ulcerative mucosal lesions
	Taste changes
Subsequent primary malignancies [19,75,76,77,78]	
**Recommendations**	
Early identification of risk factors for late oral complication	
Early diagnosis and long-term follow-up of late effects	
Multidisciplinary collaboration	
Oral hygiene instruction and biofilm control (fluoride use/chlorhexidine rinse/diet counseling)	
Regular dental checkups every 3–6 months	

## Data Availability

No new data were created or analyzed in this study. Data sharing does not apply to this article.
